# Prominent Steatosis with Hypermetabolism of the Cell Line Permissive for Years of Infection with Hepatitis C Virus

**DOI:** 10.1371/journal.pone.0094460

**Published:** 2014-04-09

**Authors:** Kazuo Sugiyama, Hirotoshi Ebinuma, Nobuhiro Nakamoto, Noriko Sakasegawa, Yuko Murakami, Po-sung Chu, Shingo Usui, Yuka Ishibashi, Yuko Wakayama, Nobuhito Taniki, Hiroko Murata, Yoshimasa Saito, Masayoshi Fukasawa, Kyoko Saito, Yoshiyuki Yamagishi, Takaji Wakita, Hiroshi Takaku, Toshifumi Hibi, Hidetsugu Saito, Takanori Kanai

**Affiliations:** 1 Center for the Study of Chronic Liver Diseases, Keio University School of Medicine, Tokyo, Japan; 2 Division of Gastroenterology and Hepatology, Department of Internal Medicine, Keio University School of Medicine, Tokyo, Japan; 3 Graduate School, Keio University School of Medicine, Tokyo, Japan; 4 Division of Pharmacotherapeutics, Faculty of Pharmacy, Keio University, Tokyo, Japan; 5 Department of Biochemistry and Cell Biology, National Institute of Infectious Disease, Tokyo, Japan; 6 Virology II, National Institute of Infectious Disease, Tokyo, Japan; 7 Department of Life and Environmental Sciences, Chiba Institute of Technology, Chiba, Japan; 8 Center for Advanced IBD Research and Treatment, Kitasato Institute Hospital, Kitasato University, Tokyo, Japan; Osaka University Graduate School of Medicine, Japan

## Abstract

Most of experiments for HCV infection have been done using lytic infection systems, in which HCV-infected cells inevitably die. Here, to elucidate metabolic alteration in HCV-infected cells in a more stable condition, we established an HCV-persistently-infected cell line, designated as HPI cells. This cell line has displayed prominent steatosis and supported HCV infection for more than 2 years, which is the longest ever reported. It enabled us to analyze metabolism in the HCV-infected cells integrally combining metabolomics and expression arrays. It revealed that rate-limiting enzymes for biosynthesis of cholesterol and fatty acids were up-regulated with actual increase in cholesterol, desmosterol (cholesterol precursor) and pool of fatty acids. Notably, the pentose phosphate pathway was facilitated with marked up-regulation of glucose-6-phosphate dehydrogenase, a rete-limiting enzyme, with actual increase in NADPH. In its downstream, enzymes for purine synthesis were also up-regulated resulting in increase of purine. Contrary to common cancers, the TCA cycle was preferentially facilitated comparing to glycolysis pathway with a marked increase of most of amino acids. Interestingly, some genes controlled by nuclear factor (erythroid-derived 2)-like 2 (Nrf2), a master regulator of antioxidation and metabolism, were constitutively up-regulated in HPI cells. Knockdown of Nrf2 markedly reduced steatosis and HCV infection, indicating that Nrf2 and its target genes play important roles in metabolic alteration and HCV infection. In conclusion, HPI cell is a *bona fide* HCV-persistently-infected cell line supporting HCV infection for years. This cell line sustained prominent steatosis in a hypermetabolic status producing various metabolites. Therefore, HPI cell is a potent research tool not only for persistent HCV infection but also for liver metabolism, overcoming drawbacks of the lytic infection systems.

## Introduction

Chronic persistent infection in liver is one of the clinical characteristics of hepatitis C virus (HCV), frequently causing liver cirrhosis and hepatocellular carcinoma (HCC) [1]. Recently, in addition to the therapy of pegylated interferon plus ribavirin, emerging anti-HCV drugs are bringing about dramatic improvement for chronic hepatitis C. However, for extermination of HCV, the development of other anti-HCV drugs targeting its persistent HCV infection and a vaccine are needed.

HCV is an enveloped, positive single-stranded RNA (9.6 kb) virus belonging to the *Flaviviridae* family, and its genome encodes a large polyprotein precursor of approximately 3,000 amino acid residues, which is cleaved by host and viral proteases into ten individual proteins, *i.e*. core, envelope 1 and 2 (E1, E2), p7, and non-structural proteins (NS2, NS3, NS4A, NS4B, NS5A, and NS5B) [2], [3]. Since an infectious strain of genotype 2a HCV (JFH-1) has been established [4], *in vitro* research for HCV infection has been accelerated. We also generated an infectious strain of chimeric HCV consisting of genotypes 1b and 2a, designated as TNS2J1 strain, whose infectivity is comparable to that of JFH-1 [5] [6].

On the other hand, a hepatoma cell line, Huh7, and its subclone such as Huh7.5 are susceptible to infection with these HCV strains and have been used for *in vitro* experiments. However, the infected cells are unstable and eventually undergo cell death, so-called lytic infection. Although some cell lines persistently infected with HCV were reported, the periods of persistency were months [7]–[9]. Thus, strictly speaking, they cannot be called persistent infection systems. Here, to study HCV-infected cells in a more stable condition, we firstly established a cell line persistently infected with TNS2J1. We have maintained this cell line for more than 2 years, the longest ever reported, since the initial transfection with RNA of TNS2J.

It was noteworthy that this cell line displayed prominent steatosis, accumulation of lipid droplet (LD). Clinically, chronic hepatitis C are frequently associated with steatosis [10]. Thus, secondary, to elucidate alterations in the metabolism and gene expression underlying such steatosis, we performed integrated analyses with metabolomics and expression arrays taking advantage of the cell line established here.

Recently, it has been reported that nuclear factor (erythroid-derived 2)-like 2 (Nrf2) is a master transcriptional activator of an array of genes for metabolisms as well as genes for cytoprotection, detoxification and antioxidation [11], in complex with v-maf avian musculoaponeurotic fibrosarcoma oncogene homolog (Maf) [12]–[14]. Thus, finally, we investigated involvement of the Nrf2/Maf complex in the metabolic alteration in the HCV-persistently-infected cells.

## Results

### Establishment of an HCV-persistently-infected Cell Line, HPI Cell

**Figure 1:**
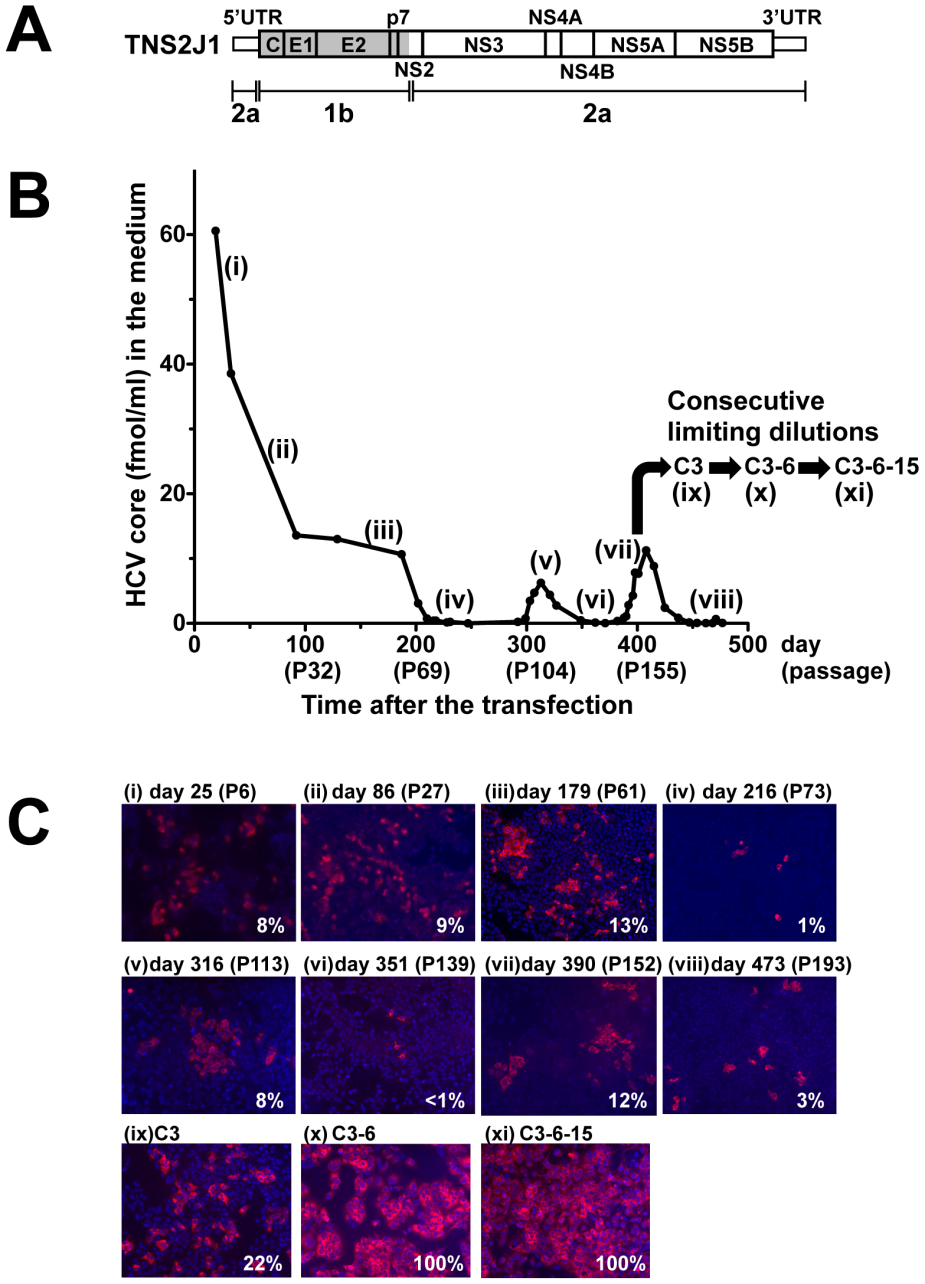
Establishment of the HCV-persistently-infected clonal cell line, HPI, monitoring HCV proteins in the culture medium and cells. (**A**) Structure of the infectious strain of a chimeric HCV (TNS2J1). Blank and shaded regions indicate genotypes 2a and 1b, respectively. (**B**) HCV core protein concentration in the medium was determined after the transfection. At time points indicated in Roman numerals, immunofluorescence staining for HCV was performed (C). Using the cells at day 396, limiting dilutions were performed three times consecutively to isolate cell clones C3 (ix), C3-6 (x), and C3-6-15 (xi). P-numbers in parentheses represent passage numbers after transfection. (**C**) Immunofluorescence staining for HCV NS5A protein in the cells was performed. Percentages indicate ration of HCV-positive cells.

We transfected Huh7.5 cells with synthetic HCV RNA of TNS2J1, where the structural region of JFH-1 (2a) was replaced with that of genotype 1b (Figure 1A) [5]. The vast majority of the infected cells with TNS2J underwent cell death, so-called ‘lytic infection’, displaying maximum of HCV core concentration in the medium (389 fmol/ml). Yet, we noticed that a tiny population of the infected cells survived this lytic phase. We maintained them for around 500 days monitoring HCV core protein concentration in the medium (Figure 1B) and checking immunofluorescence for intracellular HCV protein (Figure 1C). Even early after the transfection, at day 25 (passage 6), HCV core production was not so robust (60 fmol/ml) (i: Figure 1B), probably because the ratio of HCV-positive cells was reduced by the repeated passages and actually became undetectable at day 216 (passage 73) (i–iv: Figure 1C).

Nonetheless, we observed two minor surges of core production with slight increase in the ratio of HCV-positive cells (v and vii: Figure 1B, C) and hypothesized that the cells at the surges contain cells that are resistant to death and permissive for HCV persistent infection. In order to isolate such a cell clone, we attempted limiting dilutions using the cells at the 2nd surge (day 396). We performed this procedure three times consecutively to purify a clone, C3, C3-6 and finally C3-6-15 cell (ix, x and xi: Figure 1B, C). We considered the C3-6-15 cell as an HCV-persistently-infected cell line and designated it as ‘HPI cell’ because 100% of the cells were infected with HCV and they has supported HCV for 609 days (396 and 213 days before and during the consecutive limiting dilutions, respectively).

### HPI Cells Supported HCV Infection for More than a Year after Establishment

**Figure 2:**
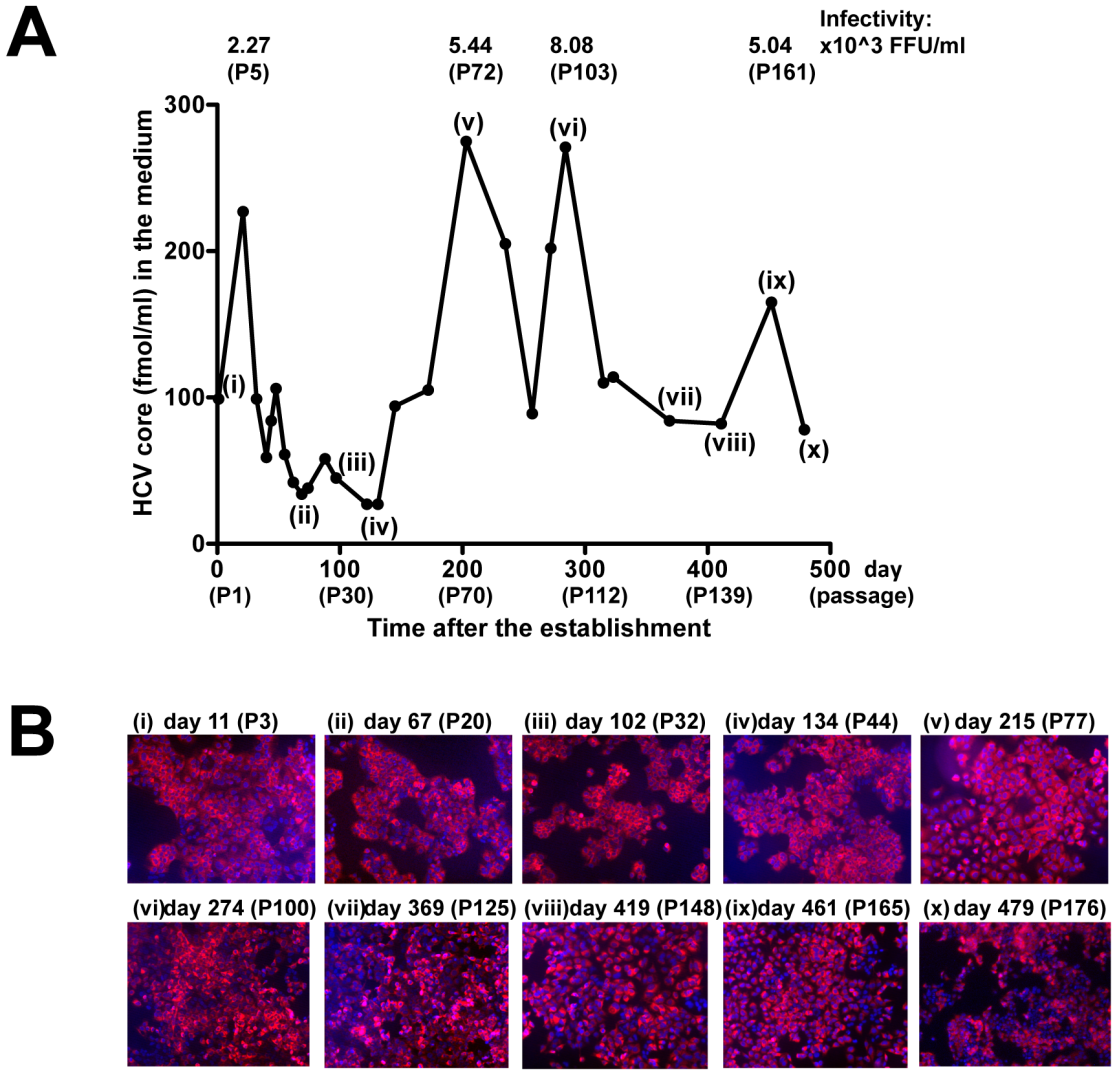
HPI cells supported HCV infection more than a year after establishment. (**A**) HCV core protein concentration in the medium was determined after the establishment of HPI cell. At time points indicated in Roman numerals, immunofluorescence staining for HCV was performed (B). Infectivity of HCVcc in the medium is shown above the graph. P-numbers in parentheses represent passage numbers after the establishment of HPI cell. (**B**) Immunofluorescence staining for HCV NS5A protein in the cells was performed.

To confirm persistence of HCV, we maintained HPI cells for about 500 days after the establishment. Core protein production was sustained all through the culture course, albeit with fluctuation from 27 to 275 fmol/ml, highest of which was comparable to that of the lytic infection (389 fmol/ml) (Figure 2A). Infectivity of HCV in cell culture medium (HCVcc) was also confirmed at passage 5, 72, 103, and 161 after the establishment, and intracellular HCV has been detected immunocytochemically at least until day 479 (Figure 2B). To ensure the existence of HCV in HPI cells, we performed RT-PCR and western blotting for HCV. PCR product covering full length of HCV, the regions from 5′-untransated region to NS2 and from NS3 to NS5A, was amplified (Figure S1A), and HCV proteins were detected in HPI cells (Figure S1B). NS5A protein of the HPI cell at passage 176 exhibited a slightly lower molecule weight than that of lytic infection and the HPI cell at passage 8. It is likely that the lower molecular weight was attributed to reduction of serine phosphorylation because deduced amino acid sequences of NS5A at passage 176 diverged remarkably and some serine residues changed to non-serine residues (manuscript in preparation). These results indicate that HPI cells have supported infectious HCV for more 479 days even after the establishment, totally for more than 2 years (1088 days) after the initial RNA transfection and that the duration is the longest ever reported to the best of our knowledge.

### Characterization of HCVcc from HPI Cells

It was shown that HCVcc from lytic infection with JFH-1 contains two types of HCV particles: low-density particles with high infectivity and high-density particles with low infectivity [15]. A similar result was obtained by sedimentation analysis of HCVcc from HPI cells (Figure S2A), suggesting that infectious HCVcc might be associated with the lipoproteins, similar to lytic infection. Then, to explore the HCV genomic variations that might have occurred in the process of the establishment, we sequenced the RT-PCR products for HCV in the culture medium of HPI cells at passage 7 and found that deduced amino acid substitutions were frequent in the E1, E2, and NS5A regions (Figure S2B).

Since the supernatant from the cultured HPI cells induced cell lysis when used to inoculate naïve Huh7.5 cells (Figure S2C), we speculated that the persistence of HCV depended not on such HCV genomic variations, but on cellular factor(s) of HPI cells. To verify this, we cured HPI cells with cyclosporine, and designated the resulting cells as ‘CuHPI’. Expectedly, these cells were susceptible to HCVcc but permissive for HCV persistency for at least 120 days (Figure S2D). Therefore, cellular factor(s), such as genetic alteration occurred during the establishment, might have conferred resistance to apoptosis and permissiveness for HCV persistent infection.

### Remarkable Accumulation of Lipid Droplets in HPI Cells

**Figure 3:**
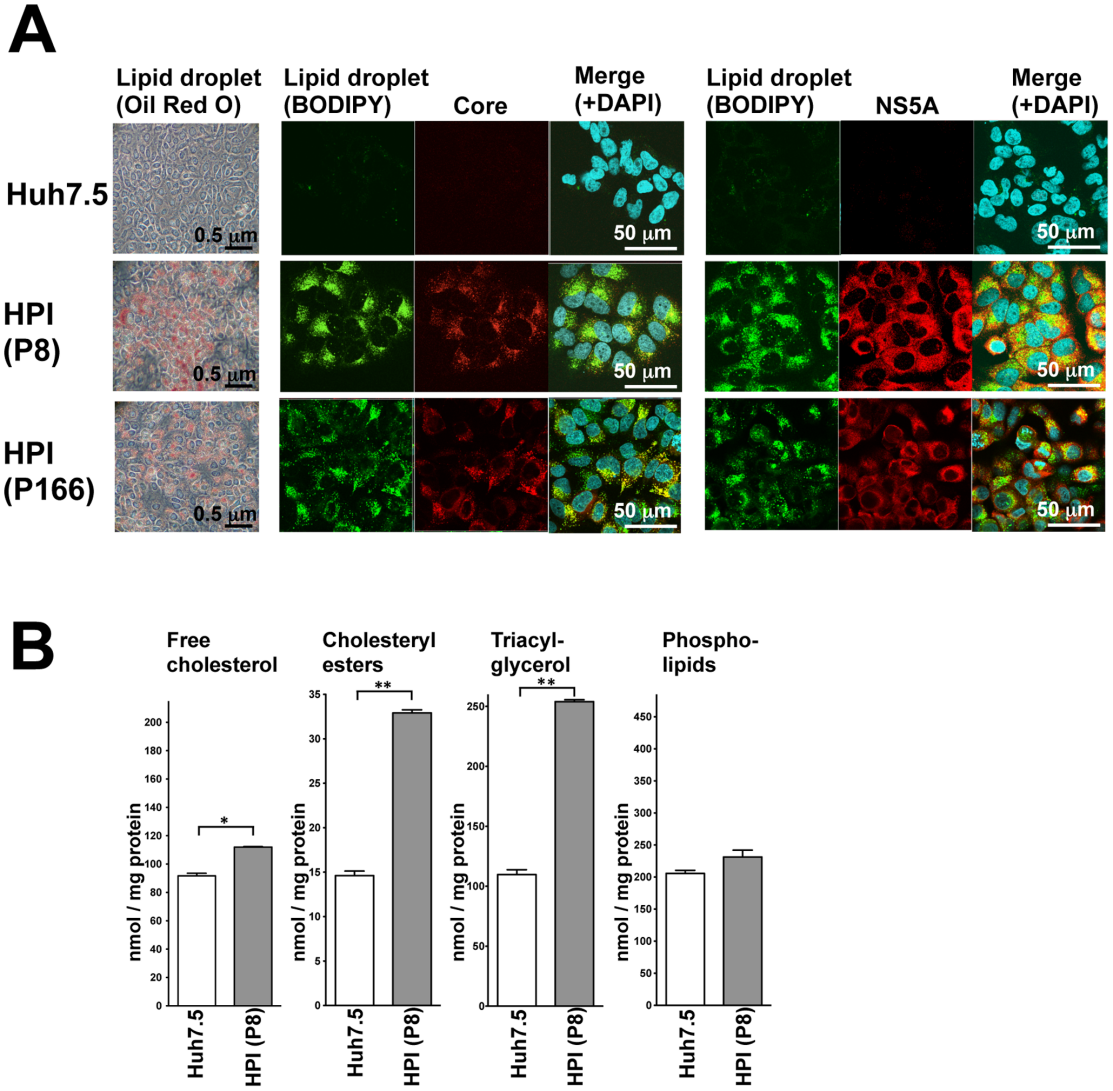
Remarkable accumulation of lipid droplets in HPI cells. (**A**) Huh7.5 cells and HPI cells at passages 8 and 166 were stained with Oil Red O for LDs (dark red) and observed with a light microscope (most left panels). They were also subjected to confocal laser scanning microscopy after immunofluorescence staining for LDs with BODIPY and for HCV core and NS5A proteins. Green fluorescence represents LDs (2nd and 5th panels from the left) and red fluorescence represents the HCV core (3rd panels from the left) and NS5A (6th panels from the left). Their images were merged with nuclei counterstained by DAPI (4th and 7th panels from the left), where the yellow areas indicate colocalization of LD with HCV proteins. The enlarged images were shown in Figure S3. (**B**) Intracellular lipid contents in Huh7.5 cells and HPI cells at passage 8 were determined in triplicate. Values were corrected by the protein concentration and statistically evaluated by Student's *t-*test indicating a significance of P<0.001 (*) and P<0.0001 (**).

It was noteworthy that prominent steatosis has sustained in HPI cells for long-term, from passage 8 to 166 as long as we observed. The core proteins were almost localized with the LDs, while the NS5A proteins were widely distributed in the cytoplasm but partly surrounding the LDs (Figure 3A, Figure S3). The distributions were similar to those of lytic infection, but the amount of LD was much more [15]. Actually, quantification of cellular lipid contents showed that major components of LDs, free cholesterol, cholesterol esters, and triacylglycerol, increased significantly in the HPI cells, whereas minor components, phospholipids, did not increase so much (Figure 3B). These result indicated HPI cell displayed prominent steatosis microscopically and biochemically.

### Integrated Analysis of HPI Cells with Metabolomics and Expression Arrays

To clarify the metabolic alteration underlying in the remarkable steatosis of HPI cells, we performed global metabolomics profiling comparing with Huh7.5 cells. For hydrophobic metabolites, liquid chromatography (LC)-time-of-flight mass spectrometry (TOFMS) was performed, and 45 metabolites were detected. Of them, the levels of 29 metabolites increased more than 1.4-fold, and five decreased to less than 0.7-fold in HPI cells (Table S1). For hydrophilic metabolites, capillary electrophoresis (CE)-TOFMS was performed, and 172 metabolites were detected. Of them, the levels of 99 metabolites increased more than 1.4-fold, and 16 decreased to less than 0.7-fold in HPI cells (Table S2). For integration of metabolomics and expression arrays, expression arrays (approximately 25,000 transcripts/array) were performed simultaneously. The expression data of genes encoding enzymes for a corresponding reaction appearing in the metabolomics profiling were selected for following pathway analyses (Table S3). Differential expression was confirmed with immunoblot analysis, when corresponding antibody was available.

### Increased Cholesterol and Desmosterol

**Figure 4:**
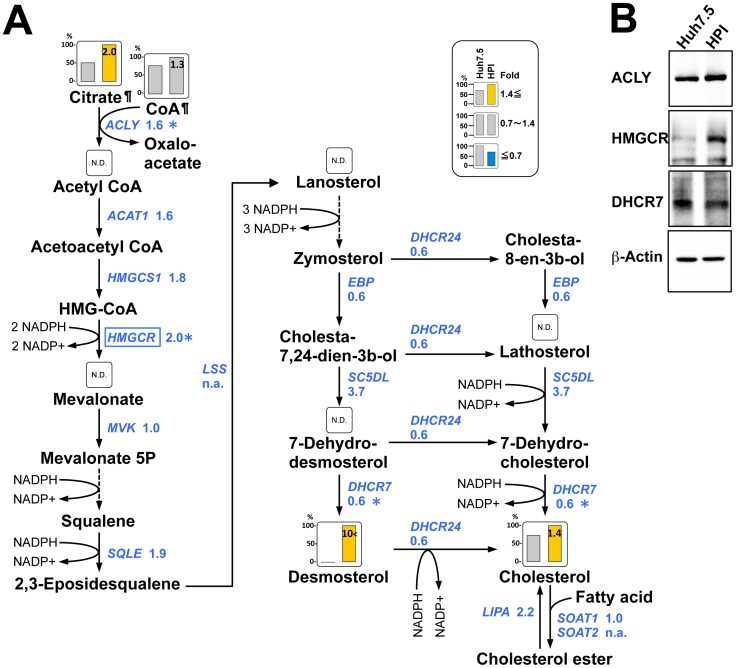
Biosynthetic pathway of cholesterol. (**A**) Relative quantities of metabolites in Huh7.5 (left column) cells and HPI cells (right column) are superimposed on a metabolic map of the cholesterol biosynthesis pathway according to the data (Table S1, Table S2). The metabolic map is depicted based on KEGG pathways (http://www.genome.jp/keg/). The NADPH/NADP+ reaction, if associated, was added to the main reaction. Height of column with larger quantity is set to 100%, and that of smaller quantity are shown proportionally. Numbers on the right columns indicate the fold-change of HPI compared to Huh7.5, and the color of the column represents: yellow (> = 1.4-fold), blue ( = <0.7-fold), or gray (0.7∼1.4-fold). ¶: A metabolite appearing on other figure. Genes (italicized) located between metabolites encode enzyme(s) for a corresponding reaction with fold-expressions of HPI to Huh7.5 according to the data (Table S3). A gene encoding a rate-limiting enzyme is surrounded by a square. *: Immunoblot analysis was also done for confirmation in (B). N.D.: Not detected. n.a.: Not assessed because of low expression. (**B**) Immunoblot analysis of the selected enzymes. Beta-actin was used as a control.

Cholesterol and cholesterol ester are major constituents in LD and HCV replication complex is fractionated in lipid raft, which is rich in cholesterol [16], [17]. Thus, at first, we analyzed the biosynthetic pathway of cholesterol. Its first step is translocation of citrate from mitochondria to cytosol, where citrate is converted to acetyl CoA. This conversion is catalyzed by ATP-citrate lyase (ACLY), whose expression was moderately up-regulated (Figure 4A, B). Increase of citrate (Figure 4A) and increase of ATP (Table 1) were noted in HPI cells. Both metabolites are known to facilitate this reaction. Moreover, a rate-limiting enzyme for this pathway is hydroxymethylglutaryl-CoA reductase (HMGCR), which was up-regulated (Figure 4A, B). In addition, other enzymes such as acetyl-CoA acetyltransferase 1 (ACAT1), hydroxymethylglutaryl-CoA synthase 1 (HMGCS1), and squalene epoxidase (SQLE) were moderately upregulated in transcription (Figure 4A), seemingly contributing to the cholesterol increase.

**Table 1:** Intracellular concentration of nucleotides.

Metabolite	Huh7.5	HPI	Fold:
	pmol/10^6^ cells	pmol/10^6^ cells	HPI/Huh7.5
**Purine metabolism**			
IMP	N.D.	N.D.	N.A.
Adenosine	3.8	6.8	1.8
Adenine	15	17	1.1
AMP	110	239	2.2
ADP	1,144	1,758	1.5
ATP	9,358	14,551	1.6
dATP	30	38	1.3
cAMP	35	25	0.7
Gaunosine	N.D.	N.D.	N.A.
GMP	44	98	2.2
GDP	335	561	1.7
GTP	1,975	3,389	1.7
**Pyrimidine metabolism**			
dTMP	N.D.	N.D.	N.A.
dTTP	85	98	1.2
Cytidine	4.7	11	2.3
CMP	32	41	1.3
CDP	123	156	1.3
CTP	2,209	2,465	1.1
dCTP	86	71	0.8
Uridine	N.D.	N.D.	N.A.
UMP	166	219	1.3
UDP	343	532	1.6
UTP	5,429	6,595	1.2

N.D.: Not detected.

N.A.: Not available.

Actual level of cholesterol was increased in the HPI cell (Figure 4A) supporting the aforementioned biochemical data (Figure 3B). Interestingly, not only an increase of cholesterol but also a drastic increase of desmosterol, a cholesterol precursor, was remarkable in HPI cells (Figure 4A). Desmosterol is converted from 7-dehydrodesmosterol by 7-dehydrocholesterol reductase (DHCR7), which was not up-regulated in HPI cell (Figure 4A, B). Rather up-regulation of sterol-5-desaturase (SC5DL) gene, upstream of DHCR7, might be attributed to the increase in desmosterol (Figure 4A). Taken together, we found that the cholesterol biosynthetic pathway was facilitated in HPI cells with up-regulation of the rate-limiting enzyme, HMGCR, and the related genes.

### Increased Fatty Acid Pool

**Figure 5:**
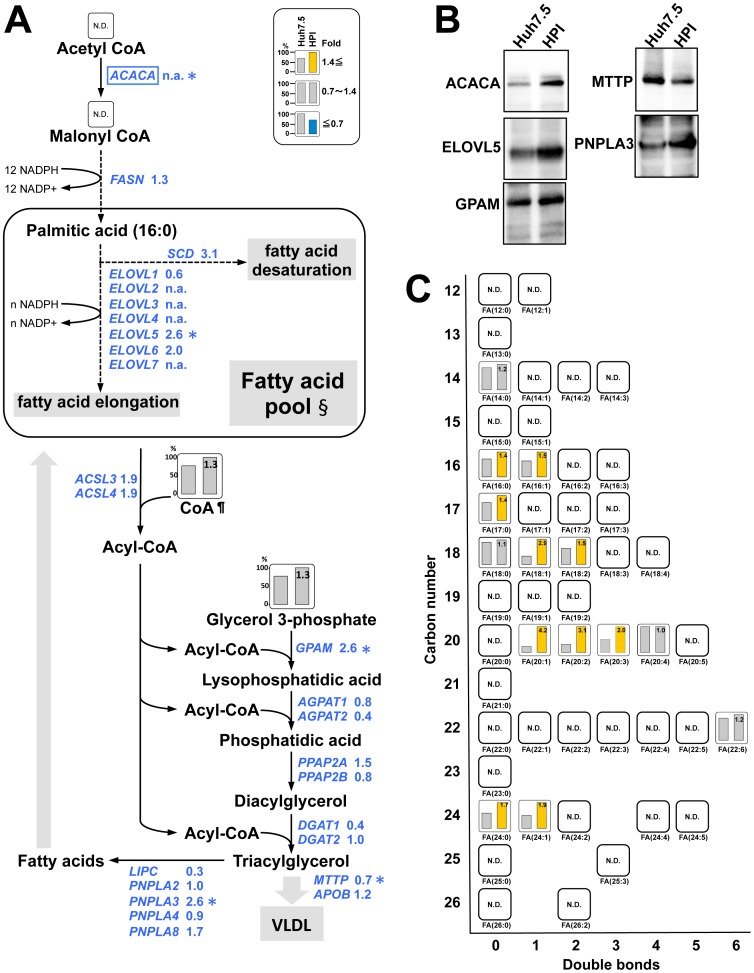
Biosynthetic pathway of fatty acids and triacylglycerol. (**A**) A metabolic map of fatty acids and triacylglycerol with gene expression is depicted in the same way as in Figure 4A. Symbols (¶, *, N.D., n.a.) represent the same meaning as in Figure 4A. §: Detailed content of individual fatty acids in the fatty acid pool are shown in (C). (**B**) Immunoblot analysis of the selected enzymes. The control was the same beta-actin as in Figure 4B. (**C**) Relative quantity of fatty acids in the fatty acid pool is represented in order of the number of carbons (Y-axis) and the number of double bonds (X-axis).

Next, we analyzed the biosynthetic pathways of fatty acids and triacylglycerol, the other major component of LD (Figure 5A). At first, cytosolic acetyl CoA is converted to malonyl CoA, and then, through multiple steps, to palmitic acid, an initial fatty acid of this pathway. Longer fatty acids are sequentially generated from palmitic acid by elongation-of-very-long-chain-fatty-acids enzymes (ELOVLs). Meanwhile, generated fatty acids are desaturated by the formation of carbon double bonds, resulting in variety of fatty acids, fatty acid pool. Up-regulation of a rate-limiting enzyme for this pathway, acetyl-CoA carboxylase α (ACACA), was noted at protein level in HPI cells (Figure 5A, B). Moderate up-regulation of the elongation enzymes, ELOVL5 and ELOVL6, and the desaturation enzymes, stearoyl-CoA desaturase (SCD), was also noted (Figure 5A). Of them, the increase of only ELOVL5 was confirmed at the protein level (Figure 5B). Resultantly, various fatty acids were remarkably increased, indicating that HPI cells were rich in fatty acid pool (Figure 5C).

In following steps, triacylglycerol is synthesized by the addition of three molecules of acyl-CoA, which are generated from fatty acids, to one molecule of glycerol 3-phosphate. The addition of the first acyl-CoA to glycerol 3-phosphate is catalyzed by glycerol-3-phosphate acyltransferase (GPAM), the expression of which was moderately up-regulated in HPI cells (Figure 5A, B). The addition of the third acyl-CoA is catalyzed by diglyceride acyltransferase (DGAT) 1 and 2. DGAT1 works predominantly for very low-density lipoprotein (VLDL) formation [18]. Thus, down-regulation of DGAT1 gene in HPI cell (Figure 5A) might have resulted in LD accumulation by reducing VLDL release. In addition, microsomal triglyceride transfer protein (MTTP) is involved in VLDL assembly and release [18] [19]. In HPI cell, down-regulation of MTTP was also noted (Figure 5A, B), probably also reducing VLDL release. On the other hand, patatin-like phospholipase domain-containing protein 3 (PNPLA3), which catabolizes triacylglycerol, was moderately up-regulated (Figure 5A, B) and hepatic lipase (LIPC), which is secreted from the cell and degrades extra-cellular triacylglycerol to free fatty acids for intake, was down-regulated (Figure 5A), suggesting negative feedback against the excess accumulation of LDs. Taken together, biosynthesis pathway of fatty acid and triacylglycerol was facilitated in HPI cells with up-regulation of the rate-limiting enzyme, ACACA, and down-regulation of genes to inhibit VLDL release.

### Facilitation of the Pentose Phosphate Pathway, Purine Synthesis Pathway, and Serine Synthesis Pathway

**Figure 6:**
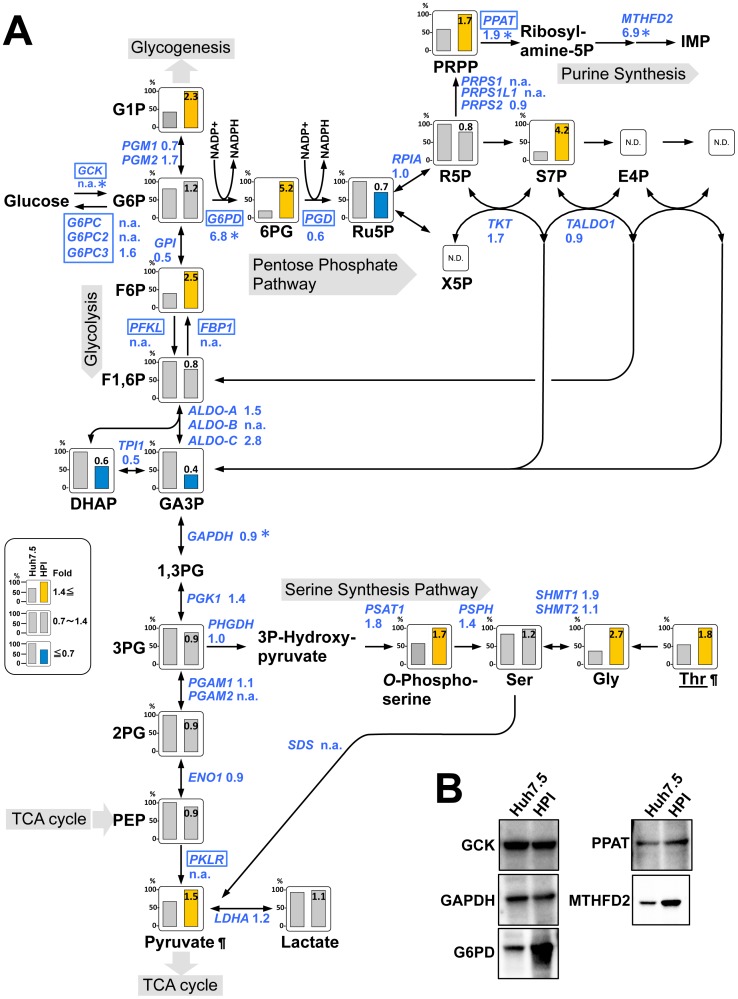
Glycolysis, pentose phosphate pathway, and serine synthetic pathway. (**A**) A metabolic map of glycolysis, the pentose phosphate pathway, and the serine synthetic pathway with gene expression is depicted in the same way as in Figure 4. Underlined: essential amino acid. Abbreviations: G1P, glucose-1-phosphate; G6P, glucose-6-phosphate; F6P, fructose 6-phosphate; F1,6P, fructose 1,6-diphosphate; DHAP, dihydroxyacetone phosphate; GA3P, glyceraldehyde 3-phosphate; 1,3PG, 1,3-Bisphosphoglycerate; 3PG, 3-Phosphoglyceric acid; 2PG, 2-phosphoglycerate; PEP, phosphoenolpyruvate; 6PG, 6-phosphogluconolactone; Ru5P, ribulose 5-phosphate; X5P, xylulose 5-phosphate; R5P, ribose 5-phosphate; S7P, sedoheptulose 7-phosphate; E4P, erythrose 4-phosphate; X5P, xylulose 5-phosphate; PRPP, phosphoribosyl pyrophosphate; IMP, inosine monophosphate; Ser, serine; Gly, glycine; Thr, threonine. Symbols (¶, *, N.D., n.a.) represent the same meaning as in Figure 4A. (**B**) Immunoblot analysis of the selected enzymes. The control was the same beta-actin as in Figure 4B.

Then we analyzed the glycolysis pathway because it is at a center of metabolisms followed by the tricarboxylic acid (TCA) cycle. As a first step, extracellular glucose is taken up into a cell and is converted to glucose-6-phosphate (G6P), whose level did not increase much in HPI cells (Figure 6A). Nor, glucokinase (GCK), a rate-limiting enzyme for this step, was not up-regulated at least in the protein level (Figure 6B). Nonetheless, three immediate intermediates of G6P increased remarkably: glucose-1-phosphate, 6-phosphogluconic acid, and fructose 6-phosphate, which lead to glycogenesis, pentose phosphate pathway (PPP), and glycolysis, respectively (Figure 5A). Level of most metabolites in glycolysis did not change or even decreased except for increase in pyruvate, a final product (Figure 6A). Expression of glyceraldehyde-3-phosphate dehydrogenase (GAPDH) did not differ (Figure 6A, B) as often used as a control for expression analysis. These results indicated glycolysis pathway was not facilitated in HPI cells as a whole.

PPP, an alternative pathway of glycolysis, supplies NADPH for reductive biosynthesis of cholesterol and fatty acids as well as for reduction of reactive oxygen species (ROS), and also supplies pentose for the following purine synthesis. In HPI cells, 6-phosphogluconate (6PG), an initial intermediate of the PPP, and sedoheptulose-7-phosphate (S7P) were remarkably increased (Figure 6A). Glucose-6-phosphate dehydrogenase (G6PD) is a rate-limiting enzyme for the PPP to catalyze G6P to 6PG in association with NADPH production. Of note, the G6PD was up-regulated drastically (6.8-fold) (Figure 6A, B), and actual level of NADPH was increased in HPI cells (Table 2).

**Table 2:** Intracellular concentration of NAD(P)+ and NAD(P)H.

Metabolite	Huh7.5	HPI	Fold:
	pmol/10^6^ cells	pmol/10^6^ cells	HPI/Huh7.5
NAD+	752	1500	2.0
NADH	(3.1E-03)	(2.4E-03)	0.8
NADP+	110	132	1.2
NADPH	(6.1E-03)	(9.7E-03)	1.6

Numeral in a parenthesis indicates relative area by the CE-FOFMS.

In purine synthesis pathway, ribose 5-P (R5P) is synthesized through the PPP and converted to phosphoribosyl pyrophosphate (PRPP), which is catabolized to inosine monophosphate (IMP) and finally to purines, AMP and GMP. In HPI cells, the level of PRPP was increased with up-regulation of phosphoribosyl pyrophosphate amidotransferase (PPAT), its rate-limiting enzyme, and methylenetetrahydrofolate dehydrogenase 2 (MTHFD2) (Figure 6A, B). In fact, the level of AMP and GMP increased remarkably together with an increase in other purines; ADP, ATP, GDP, and GTP (Table 1). Moreover, *o*-phosphoserine, glycine, and threonine in serine synthetic pathway, another alternative pathway of glycolysis, were remarkably increased in HPI cell, although the increase of serine *per se* was small (Figure 6A). Taken together, although glycolysis pathway was not facilitated, its alternative pathways including the pentose phosphate pathway, purine synthesis, and serine synthesis were facilitated in HPI with actual increase in NADPH and purines.

### Facilitation of the TCA Cycle and Increase in Amino Acids

**Figure 7:**
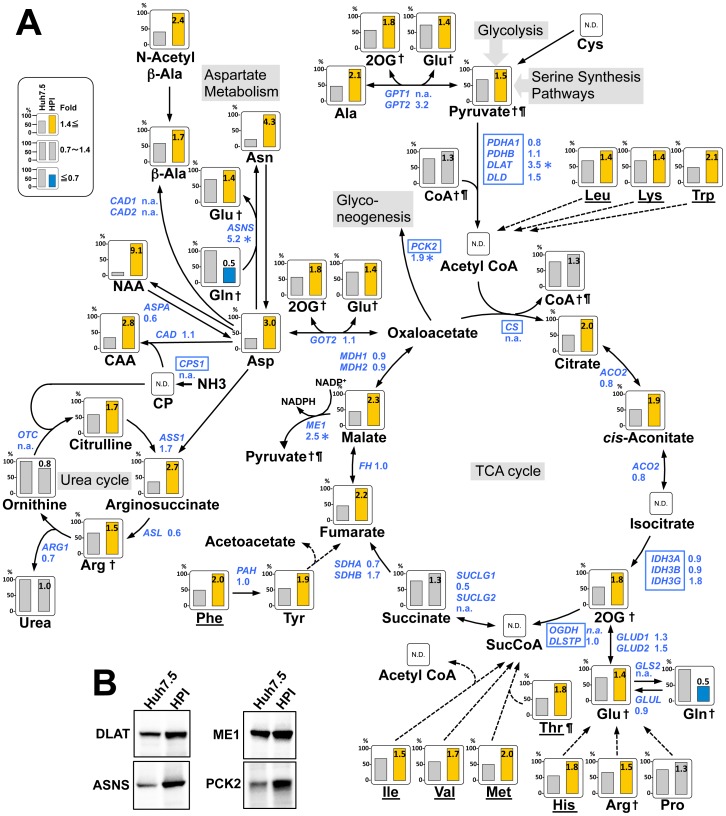
TCA cycle, amino acid metabolism, and the urea cycle. (**A**) A metabolic map of the TCA cycle, amino acid metabolism, and the urea cycle with gene expression is depicted in the same way as in Figure 4. Underlined: essential amino acid. †: Metabolite appearing more than twice in this figure. Abbreviations: Cys, cysteine; Ala, alanine; Glu, glutamate; 2OG, 2-oxoglutarate; Leu, leucine; Lys, lysine; Trp, tryptophan; Gln, glutamine; Pro, proline; Arg, arginine; His, histidine; Met, methionine; Val, valine; Ile, isoleucine; Phe, phenylalanine; Tyr, tyrosine; Asp, aspartate; NAA, N-acetylaspartate; CAA, N-carbamoyl aspartate; CP, carbamoyl phosphate; NH3, ammonia. Symbols (¶, *, N.D., n.a.) represent the same meaning as in Figure 4A. (**B**) Immunoblot analysis of the selected enzymes. The control was the same beta-actin as in Figure 4B.

Cytosolic pyruvate is transferred into the mitochondria, and, by the action of the pyruvate dehydrogenase complex, is converted to acetyl-CoA as a starting material for the TCA cycle. One subunit of this complex, dihydrolipoamide S-acetyltransferase (DLAT), was up-regulated in HPI cells (Figure 7A, B). As a whole, TCA cycle was facilitated with remarkable increase in citrate, *cis*-aconitate, 2-oxyglutarate, fumarate, and malate (Figure 7A). TCA cycle produces most of ATPs, actual level of which was increased in HPI cells (Table 1). Interestingly, phosphoenolpyruvate carboxykinase 2 (PCK2) gene, a rate-limiting enzyme for gluconeogenesis, was up-regulated in HPI cell (Figure 7A, B) and actually we confirmed increased glucose release from HPI cell (data not shown). Moreover, the expression of ME1, which produces NADPH, from malate in TCA cycle, was up-regulated in HPI cells (Figure 7A, B), suggesting contribution of ME1 in NADPH production together with the facilitated PPP.

Surprisingly, most of the essential amino acids were elevated; leucine, lysine, and tryptophan, histidine, isoleucine, valine, methionine, threonine, and phenylalanine (Figure 7A), speculating increased uptake of amino acids form outside of the cells since not all of them can be synthesized in human cells. Levels of non-essential amino acids, i.e., glutamate, tyrosine, aspartate (Asp), asparagine (Asn), arginine and alanine, were remarkably elevated in HPI cells, with only exception of decrease in glutamine (Figure 7A), indicating enhanced production of non-essential amino acids in association with the facilitation of TCA cycle,

Remarkable increase of Asp, which is catabolized from oxaloacetate in the TCA cycle, seems to have enhanced the urea cycle, since levels of argininosuccinate, arginine and citrulline were increased actually (Figure 7A). Increase of this amino acid might have caused also increase in N-acetylaspartate, N-carbamoyl aspartate, Asn and β-alanine (Figure 7A). Moreover, increase in Asn, converted from Asp coupled with the conversion of glutamine to glutamate, might be caused by up-regulation of its catalyzing enzyme asparagine synthetase (ASNS) (Figure 7A, B). Taken together, the TCA cycle was remarkably facilitated and maintained HPI cells in a hypermetabolic status with a marked increase in most of the amino acids and ATP.

### Transactivation of Genes under Control of the Transcription Complex Nrf2/Maf-G

**Figure 8:**
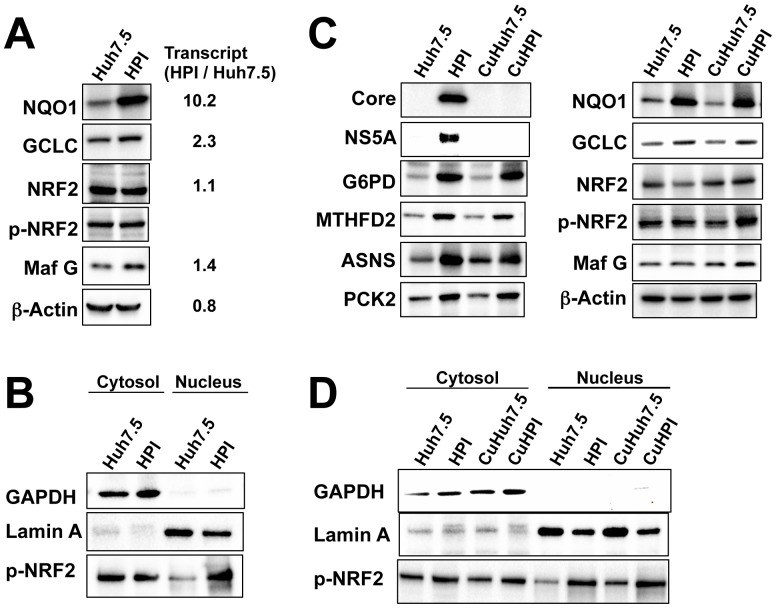
Expression of Nrf2, Maf G, and Nrf2-targert genes. (**A**) Immunoblot analyses of the proteins related to antioxidation and detoxification, Nrf2, phosphorylated Nrf2 (p-Nrf2), and Maf G for Huh7.5 and HPI cells. Fold expression of transcript (HPI/Huh7.5) in corresponding genes was shown in the right according to the expression array data. (**B**) Immunoblot analysis of p-Nrf2 in cytosol and nucleus fractions from the cells used in (A). GAPDH and Lamin A were used as a marker protein for cytosol and nucleus, respectively. (**C**) Immunoblot analyses of the HCV core and NS5A proteins, Nrf2-trarget genes, Nrf2, p-Nrf2 and Maf G for Huh7.5 and HPI cells, CuHuh7.5 cells (Huh7.5 cells simply treated with cyclosporine) and CuHPI cells (HPI cells, from which HCV was eliminated with cyclosporine). (**D**) Immunoblot analysis of p-Nrf2 in their cytosol and nucleus fractions from the cells used in (C).

Intriguingly, of the genes up-regulated in the present study, G6PD, MTHFD2, ASNS, ME1, and PCK2 belong to an array of genes under the control by the transcription factor complex, Nrf2/Maf[11]–[14]. Therefore, additionally we examined expression of antioxidant and detoxification genes, NAD(P)H dehydrogenase quinone 1 (NQO1) and gamma-glutamylcysteine synthetase (GCLC), both of which are also targets of Nrf2. As expected, they were up-regulated in HPI cells (Figure 8A). However, Nrf2 itself, and phosphorylated Nrf2 (p-Nrf2), active form of Nrf2, were not increased, whereas Maf G, a member of the Maf family, was increased but slightly. As p-Nrf2 is translocated to nucleus, then we performed immunoblot analysis separately for cytosol and nucleus and found that amount of p-Nrf2 in nucleus was more in HPI cell (Figure 8B), indicting that p-Nrf2 increased in nuclei to transactivate its related genes.

To determine whether the Nrf2/Maf-G-controlled genes were constitutively up-regulated in HPI cell, we explored their expression in the aforementioned CuHPI cells, in which HCV had been eliminated. In spite that HCV proteins were not detected in the CuHPI cell, the Nrf2 target genes were constitutively increased in CuHPI cells regardless of the presence of HCV. Although Nrf2, p-Nrf2 and Maf G did not change much (Figure 8C), p-Nrf2 in the nucleus was increased in the CuHPI cells as observed in HPI cell (Figure 8D). These results indicate that the transactivation of the Nrf2 target genes in HPI cell might be attributed to the constitutive increase of nuclear p-Nrf2.

### Knockdown of Nrf2 Reduced Lipid Droplets and HCV

**Figure 9:**
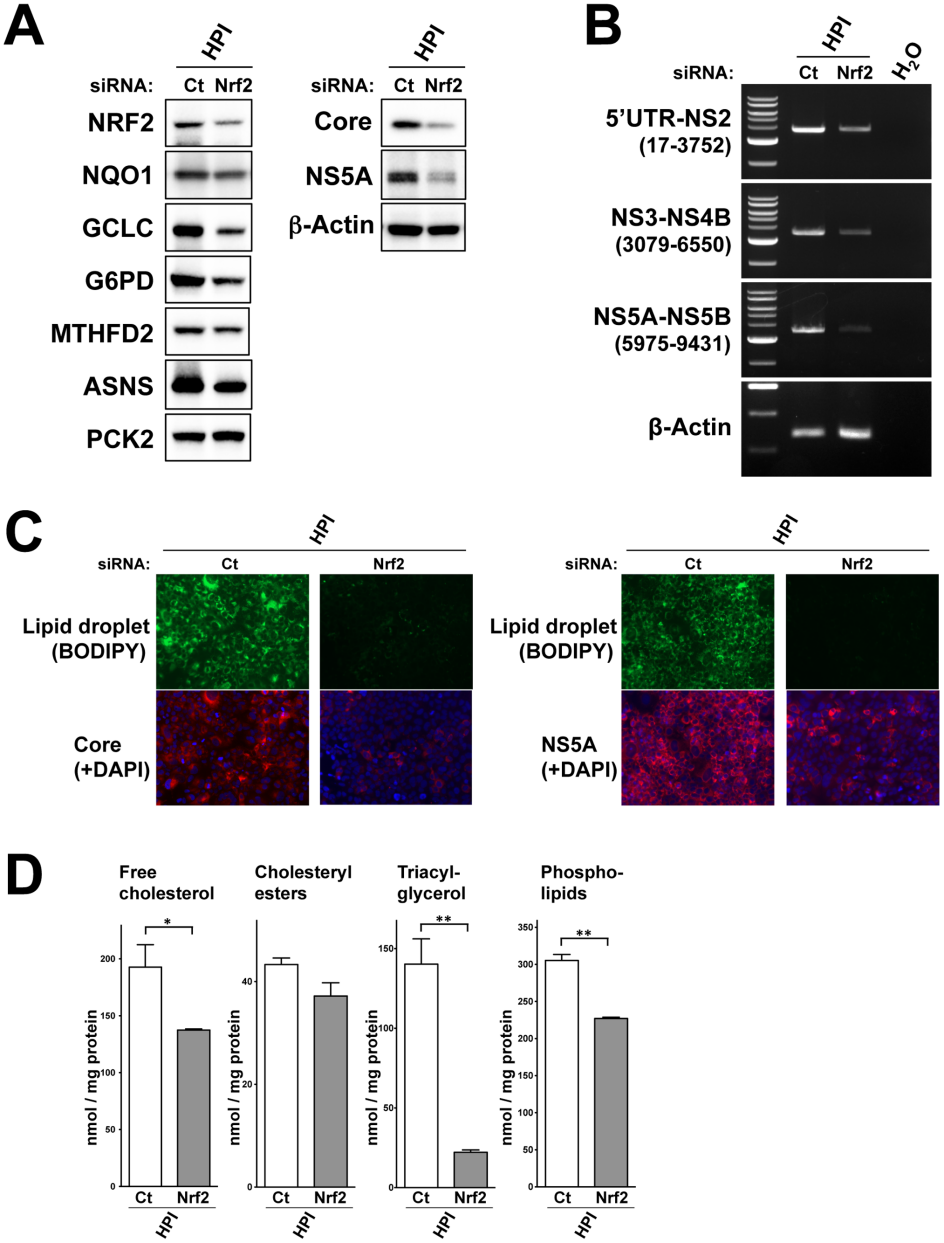
Knockdown of Nrf2 reduced lipid droplets and HCV in HPI cell. For knockdown of Nrf2, HPI cells were transfected with siRNA for Nrf2 or negative control siRNA (Ct) three times and were analyzed 2 days after the last transfection. (**A**) Immunoblot analysis of the protein for Nrf2, Nrf2-target genes and HCV (core and NS5A) was performed. (**B**) RT-PCRs were performed for three parts of HCV (5′UTR-NS2, NS3-NS4B and NS5A-NS5B) covering the full genome of HCV. Number in parenthesis indicates HCV nucleotide position amplified by RT-PCR. The most left lane of each panel represents size maker (2, 3, 4, 5, 6, 8 and 10 kb) for HCV and that for β-actin (0.2, 0.3 and 0.4 kb). (**C**) Fluorescence staining for LDs and simultaneous immunofluorescence staining for HCV core and NS5A proteins (four left panels and four right panels, respectively) were performed. Nuclei were counterstained with DAPI. (**D**) Intracellular lipid contents were determined in triplicate. Values were corrected by the protein concentration and statistically evaluated by Student's *t-*test indicating a significance of P<0.05 (*) and P<0.01 (**).

Then, to investigate whether Nrf2 affects lipid accumulation and HCV infection in HPI cell, we performed Nrf2 knockdown with small interfering RNA (siRNA). Knockdown of Nfr2 reduced expression of the target genes, NQO1, GCLC, G6PD and ASNS albeit with less extent in MTHFD2 and PCK2 (Figure 9A) in HPI cell. Importantly, the knockdown reduced HCV proteins, core and NS5A, remarkably. Reduction of HCV was verified at RNA level as well by RT-PCRs for overlapping three portions of HCV genome (Figure 9B). Moreover, fluorescence histochemistry showed that Nrf2 knockdown markedly reduced LDs and HCV infection in HPI cell (Figure 9C). Cellular lipid contents were actually reduced by the knockdown of Nrf2 (Figure 9D). Especially, reduction of triacylglycerol, main component of LD, was greatest. Collectively, these results indicate that Nrf2 and its target genes are intimately involved in the lipid metabolism of HPI cell, especially in LD formation, and persistent infection of HCV.

## Discussion

Although cell culture systems for HCV-persistent-infection have been reported, the period of persistency is months [7]–[9]. Therefore, HPI cell is a *bona fide* HCV-persistently-infected cell line because it has supported HCV for more than two years. Clinically, genotype 1b HCV is more susceptible to chronic hepatitis leading to liver cirrhosis and carcinoma, and thus an infectious strain of genotype 1b has been more required and actually established [20]. However, its infectivity is not so robust as the JFH-1. Therefore, we generated chimeric HCV, TNS2J1, and have used it for transient infection experiments [5] [6]. We started with both JFH-1 and TNS2J1 for a persistent infection system. However, JFH-1-infected cells vanished completely after 3 months, the reason why was unknown.

It was noteworthy that HPI cell has sustained steatosis as observed in chronic hepatitis C for long term. HCV modulates lipid metabolism to promote HCV replication [21], [22] and induces steatosis in its transgenic mice [23]. HCV core protein associates with LDs [15], [24]. Moreover, LDs are shown to function in the assembly and release of HCV particles dependent on apolipoprotein B expression and VLDL secretion [25]–[27]. Thus, we speculate the core protein plays a pivotal role in the development of steatosis as ‘a viral factor‘ in HPI cells.

Since HMGCR is known to increase cholesterol and inhibitors of HMGCR, statins, inhibit HCV replication [28], it may be involved also in the persistence of HCV in HPI cells. Recent reports demonstrated that miroRNA-122 (miR-122) regulates cholesterol metabolism by the down-regulation of HMGCR [29] and HCV replication [30], Moreover, deletion of miR-122 results in hepatosteatosis and tumorigenesis in mice [31]. Therefore, investigation of miroRNA including miR122 will be required for HPI cell.

In relation to cholesterol, desmosterol was increased remarkably in HPI cell. Desmosterol is an immediate precursor of cholesterol. This conversion is catalyzed by 24-dehydrocholesterol-reductase (DHCR24). A number of recent researches have shed light on unexpected and new roles of desmosterol and DHCR24 in metabolic diseases including hepatitis C [32]. Another metabolite profiling study also showed increase of desmosterol in the HCV-infected cells and demonstrated that activity of DHCR7, which catalyzes the reaction of desmosterol precursor to desmosterol, was important for HCV replication [33]. We speculate that rather up-regulation of HMGCH, rate-limiting enzyme for cholesterol, and SC5DL might enhance cholesterol synthesis pathway in the HPI cell and that down-regulation (0.6-fold) of DHCR24 might reduce the reaction of desmosterol to cholesterol resulting in the accumulation of desmosterol. Feedback inhibition of DHCR24 by lipid accumulation such as cholesterol and fatty acids is possible in HPI cell, although further study will be needed. Collectively, we strongly suggest that desmosterol could be a new biomarker for liver steatosis.

Enrichment of fatty acid pool was demonstrated in HPI cells. We speculate that was because of increase in the synthesis by the up-regulation of ACACA and/or because of decrease in the release by the down-regulation of DGAT1 and MTTP, both of which facilitate VLDL release. It is considered that down-regulation of LIPC and up-regulation of PNPLA3 might be a negative feedback reaction against the excess amount of fatty acid pool, triacylglycerol and LD. Increase of desaturated fatty acids, which have more than 2 double bonds of carbons, were observed in the HPI cell. This could be elucidated mainly by the up-regulation of SCD, an enzyme to catalyze desaturations. Since HCV core alone up-regulates SCD and accumulates NADH resulting in reductive status and enhancement of fatty acid desaturation [34], core protein might be most responsible also in HPI cell. Moreover, ethanol enhances HCV replication through lipid metabolism and elevated NADH/NAD+ [35]. Although NADH level was not elevated in the HPI cell, NADPH was more increased, indicating that NADPH might play a predominant role in the desaturation of fatty acids. Intriguingly, we observed that these cells were rich in 20-carbon fatty acids such as arachidonic acid, which is a precursor of inflammatory mediators, the so-called eicosanoids, and can cause chronic inflammation, inducing inflammatory signals around the cell[36]–[38].

The Warburg effect is the well-established theory that cancer cells preferentially utilize glycolysis [39]. However, in spite that HPI cells are originated form hepatocellular carcinoma, the TCA cycle was more activated than the glycolysis. Thus we speculate that, contrary to common cancers, hepatic cancer infected with HCV might preferentially utilize the TCA cycle in aerobic condition as previously reported [40]. In fact, hepatic cancers are vascular-rich and prone to demand more oxygen. Interestingly, alternative pathways in the glycolysis were facilitated relating to cancer promotion such as the PPP, purine synthesis pathway and the serine synthetic pathway as described previously [40] [41]. In HPI cells, level of NADPH was high, which might be synthesized not only by G6PD from the PPP and but also ME1 form the TCA cycle. In general, synthesized NADPH is utilized for reductive biosynthesis of cholesterol and fatty acids for LD formation as well as cytoprotection by reduction of reactive oxygen species, as could have occurred in HPI cells.

HPI cells were in a hypermetabolic status producing various metabolites, especially high-energy molecules such as ATP and NADPH, and accumulating energy in the form of LDs. But where did the energy come from? Increased uptake into the cell and/or decreased release from the cell could be thinkable. Cultured hepatoma cell, Huh7.5 or HPI, take in nutrients constantly from outside the cell. We have used culture medium containing 4.5 g/l glucose, which is higher than normal glucose level in human blood (1–2 g/l). Such high glucose concentration might have forced the cells to take up excess amounts of glucose. However, there is an opposite report mentioning that gluconeogenesis is promoted by HCV infection through an HCV NS5A-mediated, FoxO1-dependent signaling pathway [42]. We also noticed up-regulation of PCK2 in HPI cells. Therefore, further study on glucose metabolism will be needed to clarify these issues.

The remarkable elevation of intracellular essential amino acid indicates increased uptake of essential amino acids from the medium, whereas the elevation of non-essential amino acid levels suggests increased in intracellular *de novo* synthesis or both. For up-take of extracellular amino acids, amino acid transporters play an important role [43] and are classified into families of soluble carrier (SLC) (http://www.bioparadigms.org/slc/menu.asp) [44]. Reportedly, glucose and amino acids are increased in tumor cells to meet the increased demand for robust proliferation, for example, by the induction of SLC2A1 and SLC5A1 for glucose [45], and by the induction of SLC7A5 for amino acids [46]. In the present study, the microarray analysis showed that some genes encoding amino acid transport were up-regulated also in HPI cells (Table S4). Of them, up-regulation of SLC1A5, SLC7A1, and SLC7A11 were remarkable, possibly leading to the increase of amino acid intake (Table S4). Notably, SLC7A11, which is a cysteine transporter (xCT), confers resistance against oxidative stress and is related to multiple cancers [47].

Present study is the first to demonstrate that the metabolic genes under the control of Nrf2, such as G6PD, MTHFD2, ASNS, ME1 and PCK2, were involved in persistent infection with HCV, although some reports showed Nrf2 induces just antioxidant and detoxifying genes in HCV-infected cells [48] [49]. According to the immunoblot analyses, Maf G was increased in HPI cell, but the extent was slight. Thus we suggest that translocation of p-Nrf2, active form of Nrf2, might play a more important role in the expression of the genes, which contribute to anti-apoptosis and HCV persistence. Actually, P-Nrf2 was constitutively increased in the nucleus of HPI cell and the genes under its control were also constitutively activated. Although precise mechanism is unclear, it is speculated that some genetic or epigenetic alterations could have occurred during the long-term culture and the clonal selections affecting the Nrf2/Maf system.

Drastic reduction of LDs and lipid contents by the Nrf2 knockdown indicates that steatosis is dependent of Nrf2 in HPI cell. Additionally, we demonstrated that knockdown of Nrf2 reduced HCV infection. Reduction of its target gene expression by the Nrf2 knockdown varied, suggesting that extent of transactivation by Nrf2 and protein stability is dependent on an individual gene. Since HCV infectious cycle is closely related to lipid metabolism and LDs [15], [50], reduction of HCV by the Nrf2 knockdown might have been caused via impairment of lipid metabolism. We need to know which target gene(s) are more responsible for HCV infection and lipid metabolism.

Emerging anti-HCV drugs will bring about further improvement in sustained virological response in HCV patients. Recent study showed the risk of HCC remains even after sustained virological response [51] [52]. Genetic or epigenetic alterations that had occurred in the hepatocytes in the HCV patients for long-term chronic infection may increase HCC risk. Nrf2 and/or its target gene might be involved in candidates of such genetic alteration, because Nrf2 is activated in many cancers and would favor cell growth arrest of cancers [53], [54] Thus, genetic experiments like ultra-deep sequencing will be needed in search of such alterations in both HPI cell and clinical HCC. Moreover, Nrf2 inhibitor could be anti-HCV drug as well as anti-HCC drug, although detrimental effects on cytoprotection and detoxification must be considered.

In conclusion, we established a *bona fide* HCV-persistently-infected cell line supporting HCV for more than two years bearing prominent steatosis. Integrated analysis by metabolomics and expression arrays revealed that this cell line was in a hypermetabolic status facilitating lipid synthesis, PPP, purine synthesis, serine synthesis and TCA cycle. Transcription factor complex Nrf2/Maf-G may be involved in such a metabolic alteration. This cell line is a potent research tool not only for persistent HCV infection, but also for hepatic metabolic, connecting infection, inflammation and carcinogenesis.

## Materials and Methods

### Cell Culture, RNA Transfection and Limiting Dilution

Huh 7.5 cells were cultured in high-glucose DMEM (Life technology) supplemented with 10% fetal calf serum. The cultured cells was transfected with synthesized RNA of TNS2J1 as described previously [55]. Then, the cells were serially passaged in 1∶3 or 1∶4 splits. Concentrations of the HCV core protein in the medium were determined by using an ELISA kit (Ortho Diagnostic, Japan). Limiting dilution was performed by seeding the cultured cells on a 96-well-plate containing 0.5 cells/well.

### Preparation of Cell Cured of HCV

HCV-persistently-infected cells was cultured with the medium containing 1 μg/ml cyclosporine A (Sigma) for 3 weeks to eliminate HCV and subjected to limiting dilution to isolate cell cured of HCV. No production of HCV was confirmed by the ELISA kit for core protein in medium and by immunofluorescence staining for intracellular HCV proteins. The cured cell was designated CuHPI.

### Virus Infection, Titration, and Sedimentation Analysis

HCV infection was performed by inoculation of culture medium containing HCVcc. Determination of HCV infectivity (FFU) and sedimentation analysis for HCVcc were performed as mentioned previously [56].

### Immunofluorescence and Cytochemical Staining

For immunofluorescence staining, cells were seeded on a chamber slide. After 2 days, they were fixed with 4% paraformaldehyde, permeabilized with 0.1% Triton X-100 solution, and blocked with 5% BSA. They were incubated with primary antibodies (1 h) and then with secondary antibody (1 h). BODIPY and DAPI were used for cytochemical staining for LDs and nucleoli, respectively. Oil Red O staining (Muto pure chemicals, Japan) and crystal violet staining (Merck) was used for detection of lipid droplet and living cells, respectively.

### Extraction of Cytosol and Nucleus Fractions

Extraction of cytosol and nucleus fractions from cultured cell was conducted by using NE-PR nuclear and cytoplasmic extraction reagents (Thermo) according to the manufacture’s protocol.

### Immunoblot Analysis

For immunoblot analysis, cultured cells were harvested in lysis buffer (Pierce). After the addition of an equal volume of 2X Laemmli sample buffer (Biorad) with 5% β-mercaptoethanol, they were heat denatured (95°C, 5 min), sonicated (10 min), and then subjected to SDS-PAGE. Proteins were transferred to a PVDV membrane, Immobilon-P (Millipore), and were blocked with 5% milk powder and subsequently incubated with primary antibody. Then after incubation with horseradish peroxidase-conjugated secondary antibody for 1 h, the proteins were visualized with the ECL Prime (Amersham).

### Primary and Secondary Antibodies for Immunofluorescence Stain and Immunoblot Analysis

Primary antibodies were for HCV proteins: core (Institute of immunology), NS5A (Antiprot), NS3 (Antiprot), NS5A (Virogen), and NS5B (Antiprot), beta-Actin (Abcam), ACYL (Cell signaling), HMGCR (Atlas antibodies), DHCR7 (Abcam), ACACA (Santa Cruz), ELOVL5 (Novusbio), GPAM (Abcam), MTTP (Abcam), PNPLA3 (Abcam), GCK (Santa Cruz), GAPDH (Abcam), G6PD (Santa Cruz), PPAT (Proteintech), MTHFD2 (Proteintech), PSAT1 (Santa Cruz), SHMT1 (Abcam), G6PC3 (Santa Cruz), DLAT (Santa Cruz), IDH3G (Santa Cruz), ASNS (Santa Cruz), ME1 (Abcam), PCK2 (Abcam), GPT2 (Santa Cruz), NQO1 (Proteintech), GCLC (Abnova), Nrf2 (Santa Cruz), phosphorylated-Nrf2 (Abcam), Maf-G (Abcam), Lamin A (Santa Cruz). HRP-labeled secondary antibodies were used against mouse IgG, rabbit IgG, and goat IgG (Amersham) dependent on the primary antibodies for immunoblot. Alexa-fluor-568-labeled goat anti-mouse secondary antibody (Invitrogen) was used for immunofluorescence.

### Quantification of Cellular Lipid Contents

Cells were suspended in PBS and disrupted by sonication. Protein concentrations of whole cell homogenates were determined using a BCA protein assay kit (Pierce). Total lipids were extracted from the homogenate according to the Bligh and Dyer method. Details of Quantification of each Lipid Contents are described in the Supporting Methods. Three ml of chloroform/methanol = 1/2 (v/v) was added to 0.8 ml of the homogenate containing 250–500 μg protein to a give one phase. After 1 ml each of chloroform and PBS were added, lower chloroform phase containing lipids was separated by centrifugation. The entire lower phase was dried up under nitrogen gas and dissolved in a small volume of chloroform/methanol = 1/2 (v/v). The lipid extract was spotted on a silica gel G60 plate (Merck) and then separated by thin-layer chromatography using hexane/diethyl ether/acetic acid = 70/30/1 (v/v) as a solvent system. Free and esterified cholesterol, triacylglycerol, and phospholipids were visualized with iodine vapors and marked. Each lipid was removed from the silica gel by scraping the appropriate area from the thin-layer plate and extracted by Bligh and Dyer method.

### 1) Determination of Free and Esterified Cholesterol Contents

Free and esterified cholesterol extracted from silica gel were dried up and dissolved in 0.5 ml of acetone. Cholesterol content in the acetone extract was fluorometrically determined using enzymatic reactions as described. Briefly, an aliquot (5 μl) of the extract or cholesterol standard (up to 1 nmol) was transferred to a well of a 96-well black plate (Labsystems) containing 50 μl/well of reaction buffer (0.1 M potassium phosphate pH 7.4, 50 mM NaCl, 5 mM cholic acid, 0.1% Triton X-100). The reactions were initiated by adding 50 μl/well of the reaction buffer containing 300 μM Ampliflu red (Sigma), 2 U/ml cholesterol oxidase, 2 U/ml horseradish peroxidase, and 0.2 U/ml cholesterol esterase (for quantification of esterified cholesterol) and allowed to proceed for 30 min at 37°C. Fluorescence intensities were measured using a multi-well plate reader (FLUOstar Optima, BMG Labtech) equipped with a filter set for excitation and emission at 544 and 590 nm, respectively.

### 2) Determination of Triacylglycerol Content

Triacylglycerol extracted from silica gel was dried up and then dissolved in 0.2 ml of 2-propanol. An aliquot (10 μl) was assayed for triacylglycerol content by using Triglyceride (INT) reagent (Sigma diagnostics) with cholesteryl oleate as a standard according to the manufacturer’s protocol.

### 3) Determination of Phospholipid Content

Total phospholipids extracted from silica gel were dried up and then dissolved in 0.5 ml of chloroform/methanol = 1/2 mixture (v/v). An aliquot (100 μl) was dried up and then assayed for phosphate content as described (Lipids, 1, 85, 1966). Briefly, the dried phospholipids were digested with 140 μl of 70% perchloric acid at 180°C for 1 hr to release inorganic phosphates. The digested fraction was mixed with 800 μl of H_2_O/1.25% ammonium molybdate/10% ascorbic acid = 5/2/1 and then heated at 100°C for 5 min. Absorbance of the phosphate***-***molybdate complex at 820 nm was measured using a spectrophotometer (Shimadzu UV-1700). The amount of inorganic phosphate was determined using KH_2_PO_4_ as a standard.

### Expression Array

The Huh7.5 and HPI cells (4×10^6^ each) were plated onto 10-cm diameter dishes and cultured for 2 days. Total RNAs from these cells (approximately 80% confluence) were prepared using an RNeasy extraction kit (QIAGEN). For expression array, cDNA microarray analysis was performed with a Human Oligo Chip 25K (Toray, Japan) and analyzed by a 3D-Gene scanner 3000 (Toray). Result of the microarray was deposited in Gene Expression Omnibus (accession number: GSE52321).

### CE-TOFMS Measurement

A dish of cultured cells (10^6^ cells/sample) was used for the extraction of intracellular metabolites. The culture medium was aspirated from the dish and cells were washed twice by 5% mannitol solution (10 ml first and then 2 ml). The cells were then treated with 800 μl of methanol and left to rest for 30 s in order to inactivate enzymes. Next, the cell extract was treated with 550 μl of Milli-Q water containing internal standards (H3304-1002) and left to rest for another 30 s. The extract was obtained and centrifuged at 2,300×*g* and 4°C for 5 min, and then 800 μl of the upper aqueous layer was centrifugally filtered through a Millipore 5-kDa cutoff filter at 9,100×*g* and 4°C for 120 min to remove proteins. The filtrate was centrifugally concentrated and re-suspended in 50 μl of Milli-Q water for CE-MS analysis (Human metabolome technologies, Japan).

### LC-TOFMS Measurement

Cell cultures were prepared and washed as per above. The cells were then treated with 1,300 μl of ethanol containing an internal standard (H3304-1002) in order to inactivate enzymes. The cells were scraped from the plate, and 1,000 μl of cell lysate was mixed with 1,000 μl of Milli-Q water by ultrasonication on ice for 5 min. After the mixture solution was centrifuged at 2,300×*g* and 4°C for 5 min, the supernatant was desiccated and then dissolved with 100 μl of isopropanol/Milli-Q water for LC-MS analysis (Human metabolome technologies).

### Knockdown of Nrf2 Gene

Cultured cells were transfected with Stealth siRNA (5′-UCACUUUGCAAAGCUUUCAACCAAA-3′, 5′-UUUGGUUGAAAGCUUUGCAAAGUGA-3′, Life technology) for knockdown of Nrf2 or Negative Control Duplexes (Life technology) as a negative control by using Lipofectamine RNAiMax reagent (Life technology).

### RT-PCR

RT-PCRs were performed to amplify three parts of HCV genome; 5′UTR-NS2, NS3-NS4B and NS5A-NS5B, with primers listed on Table S5. Reverse transcription was done at 55°C for an hour with Superscript III (Life technology). Polymerase chain reaction was done with KOD plus (Toyobo, Japan) by 18 cycles of a reaction (98°C denaturing for 10 seconds, 60°C annealing for 30 seconds and 68°C extension for 3.5 minutes).

## Supporting Information

Figure S1 **Long-term existence of HCV in HPI cells. (A)** RT-PCR for the 5′UTR-NS2 region and the NS3-NS5B region of HCV were performed using total RNA from Huh7.5 cells, TNS2J1-infected Huh7.5 cells (lytic phase), and HPI cells (passages 8 and 176). **(B)** Immunoblot analyses of the HCV core, NS3, NS5A, and NS5B were performed using cellular proteins from the same cells as in (A).
(TIF)

Figure S2 **Characterization of HCVcc from HPI cells. (A)** Sedimentation analysis of HCVcc from HPI cells. Left and right y-axes represent HCV core protein concentration (filled circles) and infectivity (filled squares) forming peaks at buoyant densities of 1.117 mg/ml and 1.108 mg/ml, respectively. **(B)** Clonal sequencing of the RT-PCR products (the 5′UTR-NS2 and NS2-NS5B regions) from HCVcc. Deduced amino acid sequences from three clones were compared with the original TNS2J1 sequence, indicating consensus and non-consensus alterations shown by black and gray vertical lines, respectively. Amino acid number corresponds to that of TNS2J1. **(C)** Naïve Huh7.5 cells were inoculated with the culture medium from HPI cells or mock. At day 2 after inoculation, immunofluorescence staining for HCV NS5A protein was performed (the most left upper and lower panels). From this point, every time the mock-transfected cells became confluent, both transfected cell cultures were split (1∶4) into two wells of a 6-well plate simultaneously. One well was used for maintaining the cell culture whereas the other was used for crystal violet staining (living cell stain) after the transfection (three upper right and three lower right panels). P-numbers in parentheses represent the passage numbers after transfection. **(D)** A cured cell clone, CuHPI, was inoculated with the supernatant from the cultured HPI cells at a MOI of 0.02 FFU/cell and maintained monitoring HCV core protein in the medium and checking intracellular HCV 5A protein by immunocytochemistry.
(TIF)

Figure S3 **Enlarged images of lipid droplets and colocalizing HCV proteins.** The merged images of confocal laser scanning microscopy for the HPI cells at passage 8 (middle panels of 4th and 7th from the left in Figure 3A) were enlarged to show colocalization of LDs with HCV core (left) and NS5A (right).
(TIF)

Table S1 Intracellular metabolites detected by LC-TOFMS.
(XLSX)

Table S2 Intracellular metabolites detected by CE-TOFMS.
(XLSX)

Table S3 Expression array data of genes encoding enzymes in metabolomics profiling.
(XLSX)

Table S4 Expression of genes coding an amino acid transporter.
(XLSX)

Table S5 Primer List for RT-PCR.
(XLSX)
