# Supplementary figures and images for: Prominent Steatosis with Hypermetabolism of the Cell Line Permissive for Years of Infection with Hepatitis C Virus
Source: PLoS One. 2014 Apr 9;9(4):e94460. doi: 10.1371/journal.pone.0094460 (PMC3981821; doi:10.1371/journal.pone.0094460)

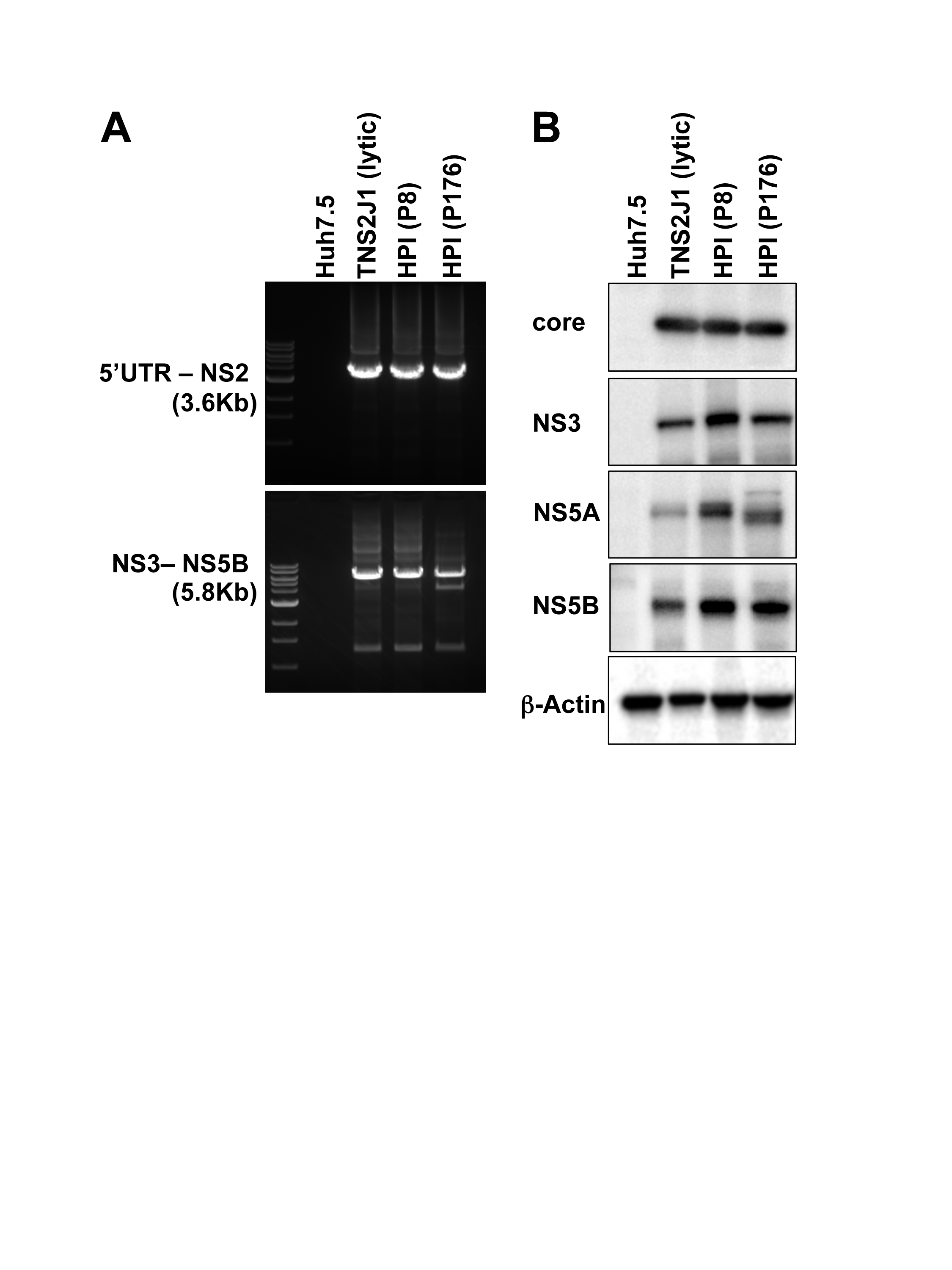

Supplement: Figure S1 — Long-term existence of HCV in HPI cells. (A) RT-PCR for the 5′UTR-NS2 region and the NS3-NS5B region of HCV were performed using total RNA from Huh7.5 cells, TNS2J1-infected Huh7.5 cells (lytic phase), and HPI cells (passages 8 and 176). (B) Immunoblot analyses of the HCV core, NS3, NS5A, and NS5B were performed using cellular proteins from the same cells as in (A). (TIF) [file pone.0094460.s001.tif]

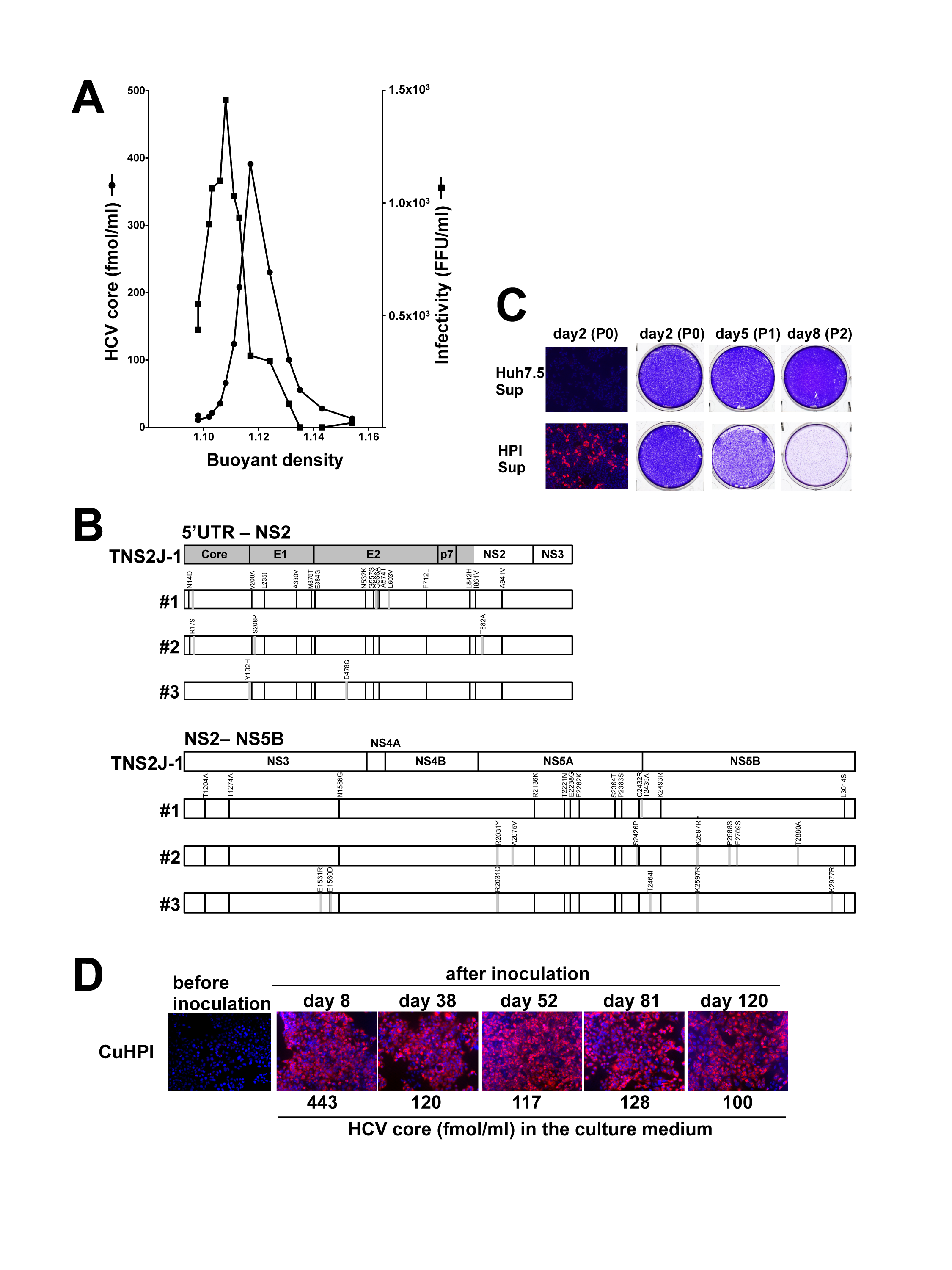

Supplement: Figure S2 — Characterization of HCVcc from HPI cells. (A) Sedimentation analysis of HCVcc from HPI cells. Left and right y-axes represent HCV core protein concentration (filled circles) and infectivity (filled squares) forming peaks at buoyant densities of 1.117 mg/ml and 1.108 mg/ml, respectively. (B) Clonal sequencing of the RT-PCR products (the 5′UTR-NS2 and NS2-NS5B regions) from HCVcc. Deduced amino acid sequences from three clones were compared with the original TNS2J1 sequence, indicating consensus and non-consensus alterations shown by black and gray vertical lines, respectively. Amino acid number corresponds to that of TNS2J1. (C) Naïve Huh7.5 cells were inoculated with the culture medium from HPI cells or mock. At day 2 after inoculation, immunofluorescence staining for HCV NS5A protein was performed (the most left upper and lower panels). From this point, every time the mock-transfected cells became confluent, both transfected cell cultures were split (1∶4) into two wells of a 6-well plate simultaneously. One well was used for maintaining the cell culture whereas the other was used for crystal violet staining (living cell stain) after the transfection (three upper right and three lower right panels). P-numbers in parentheses represent the passage numbers after transfection. (D) A cured cell clone, CuHPI, was inoculated with the supernatant from the cultured HPI cells at a MOI of 0.02 FFU/cell and maintained monitoring HCV core protein in the medium and checking intracellular HCV 5A protein by immunocytochemistry. (TIF) [file pone.0094460.s002.tif]

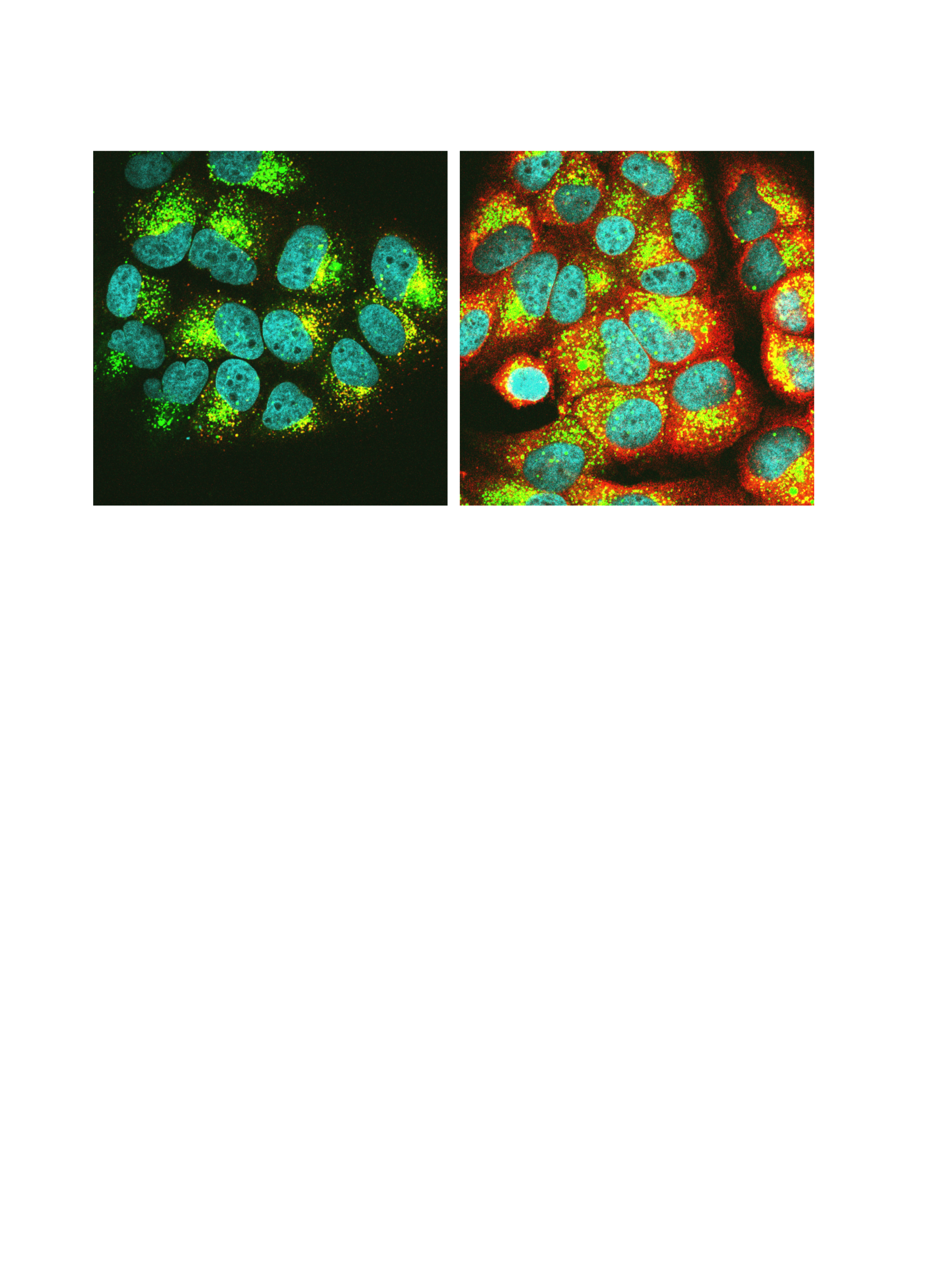

Supplement: Figure S3 — Enlarged images of lipid droplets and colocalizing HCV proteins. The merged images of confocal laser scanning microscopy for the HPI cells at passage 8 (middle panels of 4th and 7th from the left in Figure 3A) were enlarged to show colocalization of LDs with HCV core (left) and NS5A (right). (TIF) [file pone.0094460.s003.tif]
